# Supplementary material for: Rapid Discovery of Substances with Anticancer Potential from Marine Fungi Based on a One Strain–Many Compounds Strategy and UPLC-QTOF-MS
Source: Mar Drugs. 2023 Dec 18;21(12):646. doi: 10.3390/md21120646 (PMC10745104; doi:10.3390/md21120646)
Supplement: Supplementary file 1 [file marinedrugs-21-00646-s001.zip › marinedrugs-2707321-supplementary.pdf]

Table S1. Sequence alignment results of twenty-eight strains. Closest relative identification showing high sequence similarity according to NCBI GenBank by BLAST are displayed.
Table S2. Mass spectral information of differential compounds in secondary metabolites of marine fungi analysed based on UPLC-QTOF-MS technique.
Figure S1. Possible fragmentation pathway of the structure of **compounds 1-8.**

Figure S2. Possible fragmentation pathway of the structure of **compounds 9-16.**

Figure S3. Possible fragmentation pathway of the structure of **compounds 17-23.**

Table S1. Sequence alignment results of twenty-eight strains. Closest relative identification showing high sequence similarity according to NCBI GenBank by BLAST are displayed.

**Strain number**	**Sequence length**	**Accession number**	**Closest relative in NCBI GenBank**	**Sequence similarity %**	**Order**
P-WZ1	581	OK315644	*Curvularia* sp. 3 YHY-2018	100	*Curvularia* sp.
P-WZ2	562	OK315664	*Alternaria alternata* KU20017.1	100	*Alternaria* sp.
P-WZ3-1	535	OK316146	*Fusarium oxysporum* N-61-2	100	*Fusarium* sp.
P-WZ3-2	564	OK316148	*Fusarium oxysporum* R97208	100	*Fusarium* sp.
P-WZ4	545	OK316147	*Neopestalotiopsis rosae* KoRLI046308	100	*Neopestalotiopsis* sp.
P-WZ5	598	OK316891	*Pestalotiopsis* sp. Y. H. Yeh I1005	100	*Pestalotiopsis* sp.
P-WZ6	539	OK316900	*Fusarium oxysporum* N-19-2	100	*Fusarium* sp.
P-WZ10	537	OK316920	*Lasiodiplodia Pseudotheobromae* KoRLI047143	100	*Lasiodiplodia* sp.
P-WZ11	613	OK316970	*Trichoderma harzianum* ASF-T10	100	*Trichoderma* sp.
P-WZ13	540	OK317238	*Fusarium oxysporum* SF-942	100	*Fusarium* sp.
P-WS18-1	583	OK317395	*Arthrinium intestini* CBS 135835	99.13	*Arthrinium* sp.
P-WS18-2	586	OK317689	*Arthrinium* sp. GU071007	99.13	*Arthrinium* sp.
M-WS19	550	OK317702	*Aspergillus chevalieri* DTO 401-E8	100	*Aspergillus* sp.
M-WS24	624	OK318456	*Rhodotorula mucilaginosa* CBS:316	100	*Rhodotorula* sp.
M-WS25-1	602	OK319053	*Trichoderma sulphureum* SF-974	100	*Trichoderma* sp.
M-WS25-2	605	OK323230	*Trichoderma* sp. ZMQRS9	100	*Trichoderma* sp.
M-WS26	574	OK321188	*Penicillium senticosum* F1	99.83	*Penicillium* sp.
Y-WS27	605	OK323141	*Aspergillus terreus* SF-917	100	*Aspergillus* sp.
Y-WS28	548	OK323136	*Nigrospora sphaerica* 06	100	*Nigrospora* sp.
P4	545	OK323137	*Nigrospora aurantiaca* KoRLI047350	100	*Nigrospora* sp.
P3	599	OK323144	*Pestalotiopsis* sp. SC5A8	100	*Pestalotiopsis* sp.
P56	613	OK323139	*Rhodotorula mucilaginosa* CD18Y	100	*Rhodotorula* sp.
P341	602	OK323146	*Aspergillus terreus* CY229	99.83	*Aspergillus* sp.
P342	589	OK323143	*Curvularia clavata* M5	100	*Curvularia* sp.
P-L1	578	OK323138	*Talaromyces* sp. ERR11-7	100	*Talaromyces* sp.
P-L2	559	OK323242	*Fusarium* sp. YZ7-10	99.09	*Fusarium* sp.
M-L3	592	OK323142	*Diaporthe discoidispora* NKDL-2-3	99.65	*Diaporthe* sp.
M-L4	572	OK323145	*Talaromyces oumae-annae* SF-976	100	*Talaromyces* sp.

Table S2. Mass spectral information of differential compounds in secondary metabolites of marine fungi analysed based on UPLC-QTOF-MS technique.

Crude extract	NO.	Rt (min)	Mass (m/z)	ppm error	Molecular Formula	Fragment Ions (MS2)	MS2 molecular formula	Name of compound	Activity	Reference
P-WZ-2-1	**1**	9.169	706.3923	0.013	$C_{37}H_{51}N_7O_7$	237.212<br>348.179<br>371.181	$C_{14}H_{23}NO_2{}^+$<br>$C_{19}H_{30}N_3O_3{}^+$<br>$C_{19}H_{23}N_4O_4{}^+$	enamidonin	antibacterial	[1]
	**2**	14.061	462.2387	0.002	$C_{27}H_{31}N_3O_4$	252.029<br>387.214<br>444.240	$C_{17}H_{18}NO^+$<br>$C_{25}H_{27}N_2O_2{}^+$<br>$C_{26}H_{26}N_3O_4{}^+$	notoamide F	-	[2,3]
	**3**	18.047	507.3680	0.002	$C_{30}H_{50}O_6$	211.095<br>279.232<br>319.224<br>321.241	$C_{13}H_{23}O_2{}^+$<br>$C_{17}H_{27}O_3{}^+$<br>$C_{21}H_{35}O_2{}^+$<br>$C_{19}H_{29}O_4{}^+$	penicisteroid G	anticancer	[4]
	**4**	24.343	633.3997	0.068	$C_{36}H_{56}O_9$	135.115<br>163.037<br>493.402	$C_5H_{11}O_4{}^+$<br>$C_6H_{11}O_5{}^+$<br>$C_{28}H_{45}O_7{}^+$	oligoporin B	anticancer	[5]
	**5**	25.601	635.4001	0.017	$C_{32}H_{58}O_{12}$	151.035<br>363.249<br>495.416<br>563.403	$C_5H_{11}O_5{}^+$<br>$C_{16}H_{27}O_9{}^+$<br>$C_{28}H_{47}O_7{}^+$<br>$C_{27}H_{47}O_{12}{}^+$	β-D-Mannopyranoside	-	[6]
P-WZ-3-2-2	**6**	9.297	852.4893	0.022	$C_{48}H_{69}NO_{12}$	89.056<br>176.097<br>646.310	$C_4H_9O_2{}^+$<br>$C_8H_{16}O_4{}^+$<br>$C_{39}H_{52}NO_7{}^+$	chivosazole B	cytotoxic	[7]
	**7**	17.187	301.1434	0.002	$C_{18}H_{20}O_4$	121.101<br>149.093<br>180.107	$C_8H_9O^+$<br>$C_9H_9O_2{}^+$<br>$C_{10}H_{12}O_3{}^+$	trichoderolide B	cytotoxic	[8]
	**8**	19.704	427.2479	0.002	$C_{26}H_{34}O_5$	109.100<br>190.019<br>302.145<br>318.140	$C_7H_9O^+$<br>$C_{12}H_{14}O_2{}^+$<br>$C_{19}H_{26}O_3{}^+$<br>$C_{19}H_{26}O_4{}^+$	cladobotric acid D	cytotoxic	[9]
P-WZ-4-4	**9**	13.358	274.2125	0.062	$C_{13}H_{27}N_3O_3$	88.075<br>102.091<br>106.086	$C_2H_6N_3O^+$<br>$C_5H_{12}NO^+$<br>$C_{10}H_{18}N_3O_2{}^+$	fragin	anticancer	[10-12]
	**10**	13.514	318.3003	0.001	$C_{18}H_{39}NO_3$	102.090<br>132.101<br>256.263<br>257.265	$C_4H_8NO_2{}^+$<br>$C_6H_{14}NO_2{}^+$<br>$C_{15}H_{28}O_3{}^+$<br>$C_{14}H_{27}NO_3{}^+$	phytosphingosine	anticancer	[13,14]
	**11**	15.243	453.1544	0.013	$C_{25}H_{24}O_8$	119.085<br>135.080<br>147.065	$C_8H_7O^+$<br>$C_8H_7O_2{}^+$<br>$C_9H_7O_2{}^+$	thielavin S	antibacterial	[15]
	**12**	18.53	312.2169	0.146	$C_{17}H_{29}NO_4$	58.064<br>60.081<br>71.085	$C_2H_4NO^+$<br>$C_3H_8O^+$<br>$C_5H_{11}{}^+$	penicillenol D	antibacterial	[16]

	**13**	20.834	512.4256	0.08	$C_{22}H_{49}N_{13}O$	102.091<br>256.200<br>257.154<br>283.263	$C_4H_{12}N_3^{+}$<br>$C_{11}H_{26}N_7^{+}$<br>$C_{11}H_{25}N_6O^{+}$<br>$C_{13}H_{29}N_7^{+}$	cabanillasin	antibacterial	[17]
	**14**	21.354	540.4986	0.037	$C_{33}H_{65}NO$	102.091<br>256.263<br>257.266<br>311.295<br>312.235	$C_4H_8NO_2^{+}$<br>$C_{16}H_{32}O_2^{+}$<br>$C_{16}H_{33}O_2^{+}$<br>$C_{19}H_{37}NO_2^{+}$<br>$C_{18}H_{34}NO_3^{+}$	suillumide	cytotoxic	[18]
P-WZ-5-3	**15**	9.768	563.2764	0.007	$C_{21}H_{38}N_8O_{10}$	147.117<br>216.109<br>435.252<br>445.233	$C_5H_{11}N_2O_3^{+}$<br>$C_7H_{12}N_4O_4^{+}$<br>$C_{15}H_{27}N_6O_9^{+}$<br>$C_{17}H_{31}N_7O_7^{+}$	(+) -(2S,3S,4R)-10-De-O-carbamoyl-12-O-carbamoyl-Nβ-acetylstreptothricin F acid	-	[19]
	**16**	13.526	373.1646	0.002	$C_{21}H_{24}O_6$	109.065<br>123.043<br>147.075<br>315.156	$C_7H_9O^{+}$<br>$C_7H_7O_2^{+}$<br>$C_9H_7O_2^{+}$<br>$C_{17}H_{15}O_6^{+}$	penikellides A	-	[20,21]
	**17**	17.27	579.2912	0.002	$C_{29}H_{42}N_2O_{10}$	74.097<br>117.054<br>499.195<br>577.284	$C_3H_6O_2^{+}$<br>$C_4H_7NO_3^{+}$<br>$C_{27}H_{35}N_2O_7^{+}$<br>$C_{29}H_{41}N_2O_{10}^{+}$	(4R)-4,5-Dihydro-4-hydroxygeldanamycin	cytotoxic	[22]
	**18**	17.714	743.4338	0.015	$C_{38}H_{58}N_6O_9$	382.241<br>389.156<br>360.136	$C_{19}H_{33}N_3O_5^{+}$<br>$C_{20}H_{27}N_3O5^{+}$<br>$C_{19}H_{26}N_3O_4^{+}$	brintonamide B	-	[23]
P-56-2	**19**	10.915	334.2741	0.000	$C_{21}H_{35}NO_2$	91.054<br>228.231<br>242.212<br>272.258	$C_7H_7^{+}$<br>$C_{13}H_{26}NO_2^{+}$<br>$C_{14}H_{28}NO_2^{+}$<br>$C_{19}H_{30}N^{+}$	preussin I	-	[24]
	**20**	17.175	279.1590	0.002	$C_{16}H_{22}O_4$	57.070<br>149.023<br>150.027	$C_3H_5O^{+}$<br>$C_8H_5O_3^{+}$<br>$C_8H_6O_3^{+}$	cytosporone C	cytotoxic	[25,26]
P-341-2	**21**	10.578	246.1700	0.073	$C_{12}H_{23}NO_4$	88.075<br>102.091<br>202.215<br>229.235	$C_4H_8O_2^{+}$<br>$C_5H_{10}O_2^{+}$<br>$C_9H_{16}NO_4^{+}$<br>$C_{11}H_{19}NO_4^{+}$	3-hydroxy-*N*-(1-hydroxy-3-methylpentan-2-yl)-5-oxohexanamide	-	[27]
	**22**	16.821	335.2217	0.002	$C_{20}H_{30}O_4$	113.093<br>195.10<br>251.094<br>308.200	$C_7H_{13}O^{+}$<br>$C11H15O3^{+}$<br>$C_{15}H_{23}O_3^{+}$<br>$C_{18}H_{28}O_4^{+}$	eutypellone A	-	[28]
	**23**	17.27	361.2221	0.001	$C_{18}H_{32}O_7$	129.018<br>207.062<br>211.061<br>268.123	$C_5H_5O_4^{+}$<br>$C_7H_{11}O_7^{+}$<br>$C_{13}H_{23}O_2^{+}$<br>$C_{14}H_{20}O_5^{+}$	CJ-13，982	-	[29]

References

1. Son, S.; Ko, S.K.; Kim, S.M.; Kim, E.; Kim, G.S.; Lee, B. Antibacterial cyclic lipopeptide enamidonins with an enamide-linked acyl chain from a Streptomyces species. J. Nat. Prod. 2018, 81, 2462-2469.
2. Tsukamoto, S.; Kato, H.; Samizo, M.; Nojiri, Y. Notoamides F−K, prenylated indole alkaloids isolated from a marine-derived *Aspergillus* sp. *J. Nat. Prod.* 2008, 71, 2064-2067.
3. Wang, F.; Sarotti, A.M.; Jiang, G.; Huguet-Tapia, J.C.; Zheng, S.L. Waikikiamides A-C: complex diketopiperazine dimer and diketopiperazine-polyketide hybrids from a Hawaiian marine fungal strain *Aspergillus* sp. FM242. *Org. Lett.* 2020, 22, 4408-4412.
4. Xie, C. L.; Zhang, D.; Xia, J.M.; Hu, C.C.; Lin, T. Steroids from the deep-sea-derived fungus *Penicillium granulatum* MCCC 3A00475 induced apoptosis via Retinoid X Receptor (RXR)-α pathway. *Mar. Drugs.* 2019, 17, 178.
5. Lee, I.K.; Jang, Y.W.; Yu, S.H.; Yun, B.S. New triterpene glucosides, oligoporins A-C, from *Oligoporus tephroleucus* protect DNA from Fenton reaction. *Bioorg. Med. Chem. Lett.* 2007, 17, 4906–4909.
6. Wenzel, S.C.; Müller, R. Myxobacterial natural product assembly lines: fascinating examples of curious biochemistry. *Nat Prod Rep*. 2007, 24, 1211-24.
7. Perlova, O.; Gerth, K.; Kaiser, O.; Hans, A.; Müller, R. Identification and analysis of the chivosazol biosynthetic gene cluster from the myxobacterial model strain *Sorangium cellulosum* So ce56. *J. Biotechnol.* 2006, 121, 174-191.
8. Guo, Y.W.; Gong, B.Q.; Yuan, J.; Li, H.J. l-Phenylalanine alters the privileged secondary metabolite production in the marine-derived fungus *Trichoderma erinaceum* F1-1. *J. Nat. Prod.* 2020, 83, 79-87.
9. Mitova, M.I.; Lang, G.; Blunt, J.W.; Cummings, N.J. Cladobotric acids A-F: new cytotoxic polyketides from a new zealand *Cladobotryum* sp. *J. Org. Chem.* 2006, 71, 492-497.
10. Akira, M.; Kosei, H.; Saburo,T. Fragin, a New Biologically Active Metabolite of a Pseudomonas. *Agricultural and biological chemistry.* 1969, 33, 1599-1605.
11. Tamura, S.; Murayama, A.; Hata, K. Isolation and structural elucidation of fragin, a new plant growth inhibitor produced by a Pseudomonas. *Agricultural and Biological Chemistry.* 1967, 31, 758-759.
12. Nagahara, Y.; Shinomiya, T.; Kuroda, S.; Kaneko, N. Phytosphingosine induced mitochondria-involved apoptosis. *Cancer Sci.* 2005, 96, 83-92.
13. Glenz, R.; Kaiping, A.; Göpfert, D.; Weber, H. The major plant sphingolipid long chain base phytosphingosine inhibits growth of bacterial and fungal plant pathogens. *Sci. Rep.* 2022, 12, 1081.
14. Weber, S.S.; de Souza, A.C.S.; Soares, D.C.L. Chemical profile, antimicrobial potential, and antiaggregant activity of supercritical fluid extract from Agaricus bisporus. *Chem. Pap.* 2022, 76, 6205-6214.
15. De Medeiros, L.S.; Abreu, L.M.; Nielsen, A.; Ingmer, H. Dereplication-guided isolation of depsides thielavins S-T and lecanorins D-F from the endophytic fungus *Setophoma* sp. *Phytochemistry*. 2015, 11, 1154-162.
16. Chen,L.; Zhang, Q.Q.; Hu, X. Novel cytotoxic metabolites from the marine-derived fungus *Trichoderma citrinoviride*. *Heterocycles*. 2014, 89, 189.
17. Houard, J.; Aumelas, A.; Noël, T.; Pages, S.; Givaudan, A. Cabanillasin, a new antifungal metabolite, produced by entomopathogenic *Xenorhabdus cabanillasii* JM26. *J. Antibiot.* (Tokyo). 2013, 66, 617-620
18. León, F.; Brouard, I.; Torres, F.; Quintana, J. A new ceramide from suillus luteus and its cytotoxic activity against human melanoma cells. *Chem. Biodivers.* 2008, 5, 120–125.
19. Gan, M.; Zheng, X.; Gan, L.; Guan, Y.; Hao, X. Streptothricin derivatives from *Streptomyces* sp. I08A 1776. *J. Nat. Prod.* 2011, 74, 1142-1147.
20. Luo,H.; Li, X.M.; Li, C.S. Diphenyl ether and benzophenone derivatives from the marine mangrove-derived fungus *Penicillium* sp. MA-37. *Phytochemistry Lett.* 2014, 9, 22-25.
21. Zhang, F.; Gang, D.; Li,L. Isolation, antimicrobial activity, and absolute configuration of the furylidene tetronic acid core of pestalotic acids A-G. *Org. Biomol. Chem.* 2012, 10, 5307.
22. Li, T.; Ni, S.; Jia, C.; Wang, H.; Sun, G.; Wu, L.; Gan, M. Identification of 4,5-dihydro-4-hydroxygeldanamycins as shunt products of geldanamycin biosynthesis. *J. Nat. Prod.* 2012, 75,1480-1484.
23. Al-Awadhi, F.H.; Gao, B.; Rezaei, M.A.; Kwan, J.C.; Li, C. Discovery, synthesis, pharmacological profiling, and biological characterization of brintonamides A-E, novel dual protease and GPCR modulators from a marine Cyanobacterium. *J. Med. Chem*. 2018, 61, 6364-6378.
24. Gu, B.B.; Jiao, F.R.; Wu, W.; Jiao, W.H.; Li, L.; Sun, F. Preussins with Inhibition of IL-6 expression from *Aspergillus flocculosus* 16D-1, a fungus isolated from the marine sponge phakellia fusca. *J. Nat. Prod.* 2018, 81, 2275-2281.
25. Liu, H.X.; Li, H.H.; Chen, Y.C. Cytotoxic secondary metabolites from an endophytic fungal strain of phomopsis phyllanthicola. *Chinese J. Org. Chem.* 2019, 39, 1475.

26. Lena,H.; Debbab, A.; Ngoc, T.D. Polyketides from the mangrove-derived endophytic fungus Acremonium strictum. *Tetrahedron Lett*. 2014, 55, 3463-3468.
27. Xiao, J.; Zhang, Q.; Gao, Y.Q. Secondary metabolites from the endophytic *Botryosphaeria dothidea* of melia azedarach and their antifungal, antibacterial, antioxidant, and cytotoxic activities. *J. Agric. Food Chem.* 2014, 62, 3584–3590.
28. Isaka, M.; Palasarn, S.; Prathumpai, W.; Laksanacharoen, P. Pimarane diterpenes from the endophytic fungus *Eutypella* sp. BCC 13199. *Chem. Pharm. Bull.* 2011, 59,1157-1159.
29. Watanabe, S.; Hirai, H.; Kambara, T.; Kojima, Y.; Nishida, H.; Sugiura, A. CJ-13,981 and CJ-13,982, new squalene synthase inhibitors. *J. Antibiot.* (Tokyo). 2001, 54, 1025-1030.

Supplementary Figures. Possible fragmentation pathways of **compounds 1-23.**

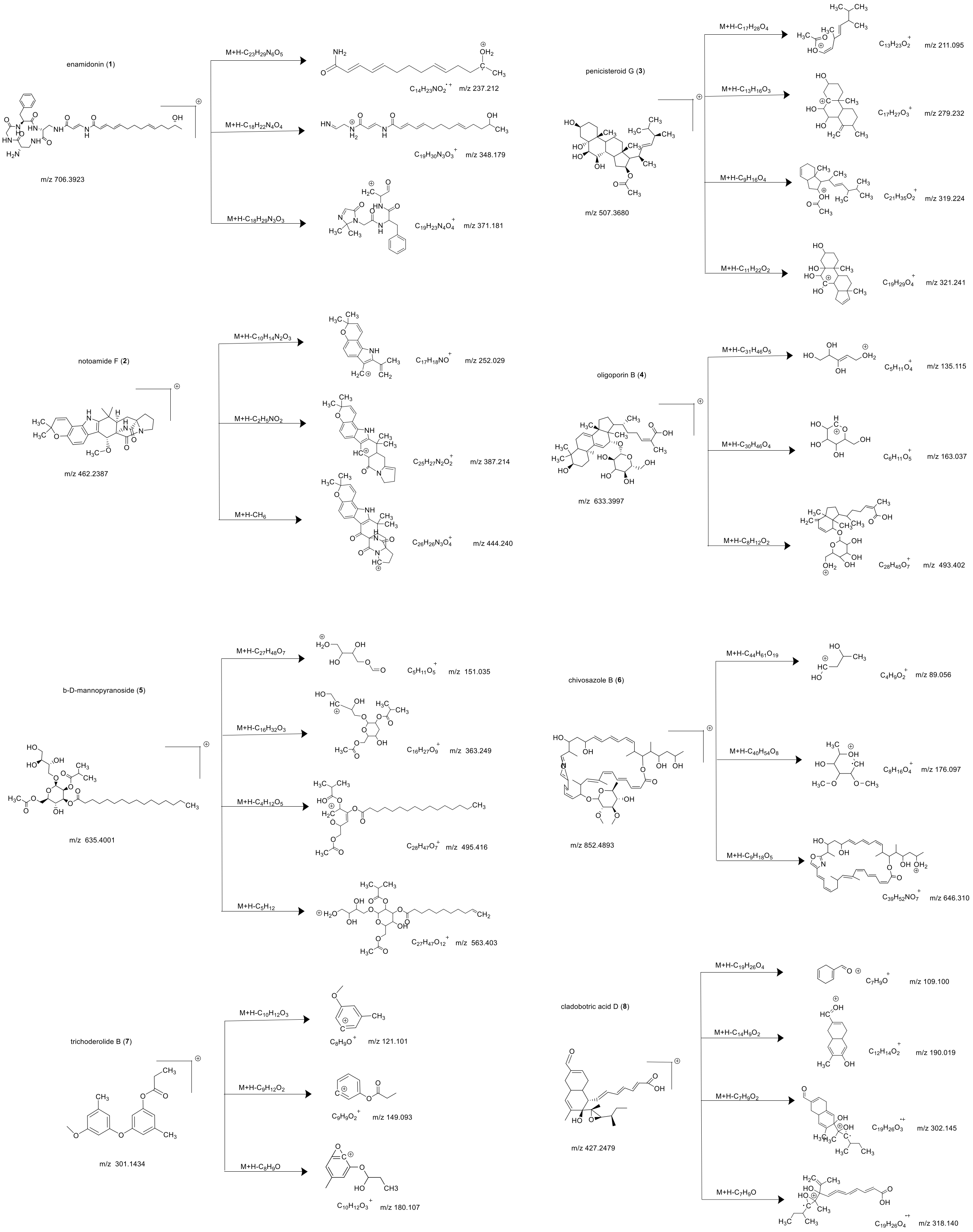

Figure S1. Possible fragmentation pathways of the structure of compounds **1-8.**

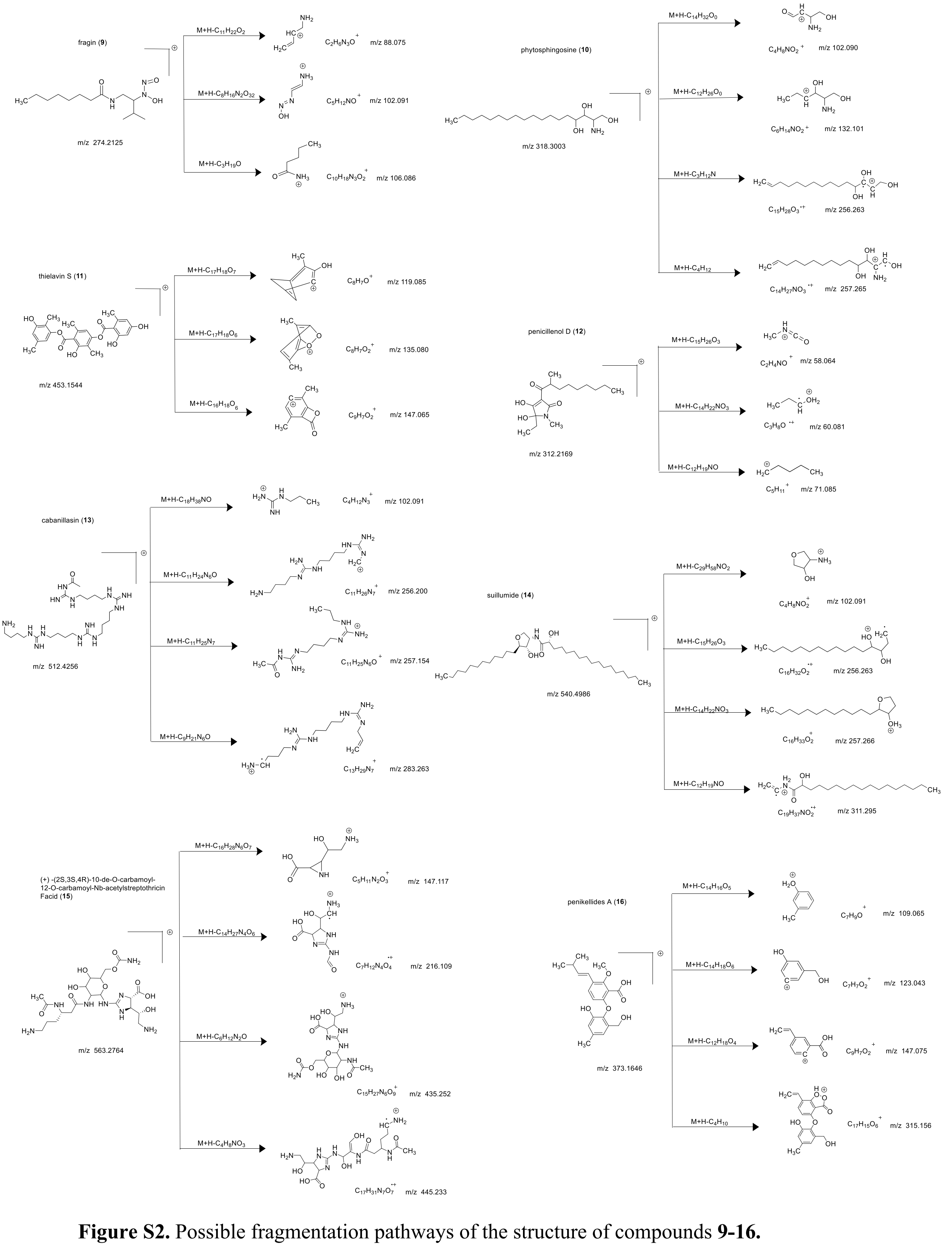

Figure S2. Possible fragmentation pathways of the structure of compounds **9-16.**

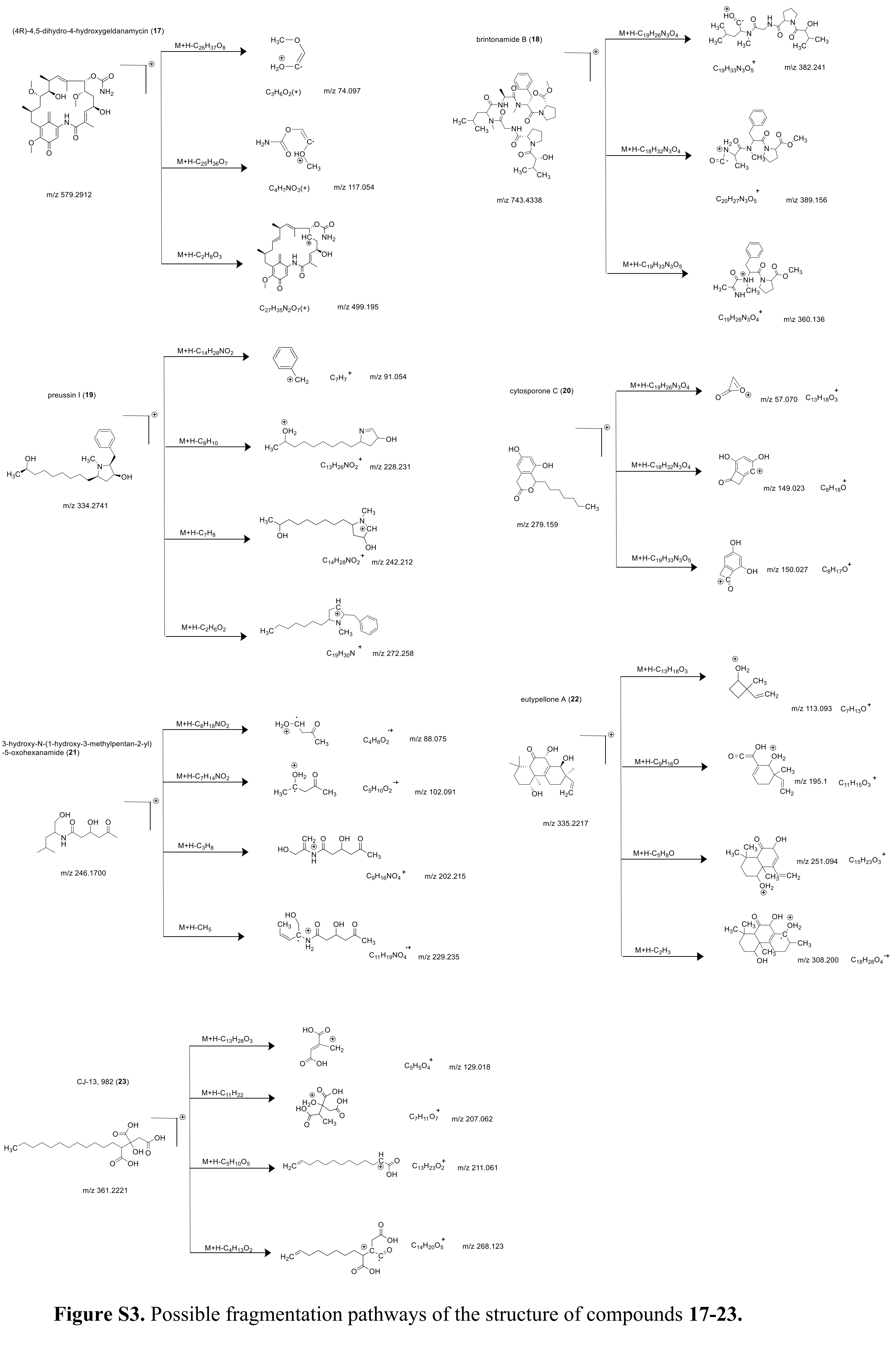

Figure S3. Possible fragmentation pathways of the structure of compounds **17-23**.
